# VRx@Home: A virtual reality at‐home intervention for persons living with dementia and their family caregivers

**DOI:** 10.1002/alz70858_103604

**Published:** 2025-12-26

**Authors:** Raheleh Saryazdi, Lou‐Anne Carsault, Julie Bannon, Erica Dove, Marie Y. Savundranayagam, Lora Appel, Jennifer L. Campos

**Affiliations:** ^1^ Trent University Durham, Oshawa, ON, Canada; ^2^ KITE ‐ Toronto Rehabilitation Institute, University Health Network, Toronto, ON, Canada; ^3^ KITE‐University Health Network, Toronto, ON, Canada; ^4^ University of California Davis, Davis, CA, USA; ^5^ KITE Research Institute, Toronto Rehabilitation Institute, University Health Network, Toronto, ON, Canada; ^6^ University of Toronto, Toronto, ON, Canada; ^7^ Western University, London, ON, Canada; ^8^ York University, Toronto, ON, Canada

## Abstract

**Background:**

Virtual Reality (VR) therapies are increasingly being evaluated for Persons Living with Dementia (PLwD), with most research revealing benefits of VR for general well‐being. VR‐therapies, however, have been assessed primarily in long‐term care or community program settings and are typically administered by formal caregivers or researchers. Comparatively little research has explored the application of VR‐therapy in an at‐home setting and for the purpose of enhancing PLwD and family caregiver interactions. The goal of this study was to evaluate whether communication between PLwD and their caregivers could be facilitated through a VR at‐home intervention (VRx@Home), and to compare VR technology to traditional Tablet‐based technology.

**Method:**

The study began with at‐home training and a baseline data collection session. Next, families first completed either 2 weeks of VR (with paired tablet for the caregiver) or 2 weeks of the Tablet‐only condition. During weekly remote sessions with a researcher, families watched 20‐min sets of videos across four themes (animals, travel, sports, entertainment), followed by completing a semi‐structured interview. They were then asked to try sessions on their own. The intervention concluded with a final interview. A comprehensive set of measures relevant to communication, technology preferences, and general feedback were collected.

**Result:**

25 PLwD and 25 caregivers participated in the intervention, with 20 families completing the entire intervention and 5 families completing a partial intervention. From those who completed the full intervention, 18 reported a preference for VR (7 PLwD, 11 caregivers), 17 for Tablet‐only (10 PLwD, 7 caregivers), and 5 for both devices equally (3 PLwD, 2 caregivers). Entertainment and animals were the favourite themes reported across all participants and all sessions. Regarding communication outcomes, our preliminary results from surveys completed by caregivers revealed that both devices improved communication (more frequent, engaging, natural, longer) compared to baseline, but VR did so to a greater extent.

**Conclusion:**

The results suggest that VR‐therapy can be implemented in an at‐home setting and has the potential to enhance communication between PLwD and caregivers. The research also highlights the variability in preferences and the importance of providing options to allow for long‐term VR adoption in at‐home settings.

